# AI-Based Protein Interaction Screening and Identification (AISID)

**DOI:** 10.3390/ijms231911685

**Published:** 2022-10-02

**Authors:** Zheng-Qing Fu, Hansen L. Sha, Bingdong Sha

**Affiliations:** 1 SER-CAT, Advanced Photon Source, Argonne National Laboratory, Argonne, IL 60439, USA; 2 Department of Biochemistry & Molecular Biology, University of Georgia, Athens, GA 30602, USA; 3 Department of Cell, Developmental and Integrative Biology (CDIB), University of Alabama at Birmingham, Birmingham, AL 35294, USA

**Keywords:** proteins binding, computer-aided screening, AlphaFold, AISID

## Abstract

In this study, we presented an AISID method extending AlphaFold-Multimer’s success in structure prediction towards identifying specific protein interactions with an optimized AISIDscore. The method was tested to identify the binding proteins in 18 human TNFSF (Tumor Necrosis Factor superfamily) members for each of 27 human TNFRSF (TNF receptor superfamily) members. For each TNFRSF member, we ranked the AISIDscore among the 18 TNFSF members. The correct pairing resulted in the highest AISIDscore for 13 out of 24 TNFRSF members which have known interactions with TNFSF members. Out of the 33 correct pairing between TNFSF and TNFRSF members, 28 pairs could be found in the top five (including 25 pairs in the top three) seats in the AISIDscore ranking. Surprisingly, the specific interactions between TNFSF10 (TNF-related apoptosis-inducing ligand, TRAIL) and its decoy receptors DcR1 and DcR2 gave the highest AISIDscore in the list, while the structures of DcR1 and DcR2 are unknown. The data strongly suggests that AlphaFold-Multimer might be a useful computational screening tool to find novel specific protein bindings. This AISID method may have broad applications in protein biochemistry, extending the application of AlphaFold far beyond structure predictions.

## 1. Introduction

Over the last several decades, various algorithms have been developed to model unknown structures based on the information deposited at the Protein Data Bank (PDB). The AphaFold2 is an Artificial Intelligent (AI) program that utilizes deep learning to perform protein structure prediction [1], which demonstrated an outstanding ability to predict the single-chain protein structures during the 14th Community Wide Experiment on the Critical Assessment of Techniques for Protein Structure Prediction (CASP14) competition [2]. Other platforms have also been developed for protein structure modeling, either independently or through exploiting the existing version of AlphaFold2 [3,4,5,6,7,8]. Lately, the AlphaFold2 team have released AlphaFold-Multimer, which added the capability to model protein complexes of multiple chains [9]. More recently, it has been shown that AlphaFold-Multimer can be utilized to predict protein complex structure with accuracy for soluble protein complexes, transmembrane ABC transporter complex, and protein complexes containing intrinsic disordered proteins (IDP) [3,10,11,12,13,14].

While precise atomic structures of protein complexes could provide insight of the detailed static interactions, identifying novel specific protein interactions may have a broader interest in protein characterizations. To identify the possible binding partner for a bait protein from a long list of protein candidates, it would be very helpful that a computational screening method can quickly predict binding potentials and prioritize the candidates for further biochemical assays. Such a method could revolutionize the protein characterization process. In this study, we presented an AI-based protein interaction screening and identification (AISID) method extending AlphaFold-Multimer’s success in structure prediction towards identifying specific protein interactions.

## 2. Results and Discussions

To predict a heterodimer protein complex structure, the AlphaFold-Multimer generates 25 (by default) complex structure models with a model confidence value for assessing the quality of each model. We derive a metric score (denoted as AISIDscore hereafter) from the model confidence values, and the AISIDscore is optimized for assessing binding potentials between the protein pairs. We hypothesize that the AISIDscore can serve as a quality indicator to evaluate the binding potential between the protein pair. In the computational screening, a bait protein and a panel of protein ligand candidates can be input into the AlphaFold-Multimer for calculations. A higher AISIDscore might indicate the higher likelihood of specific binding between the bait and the protein ligand.

**Figure 1:**
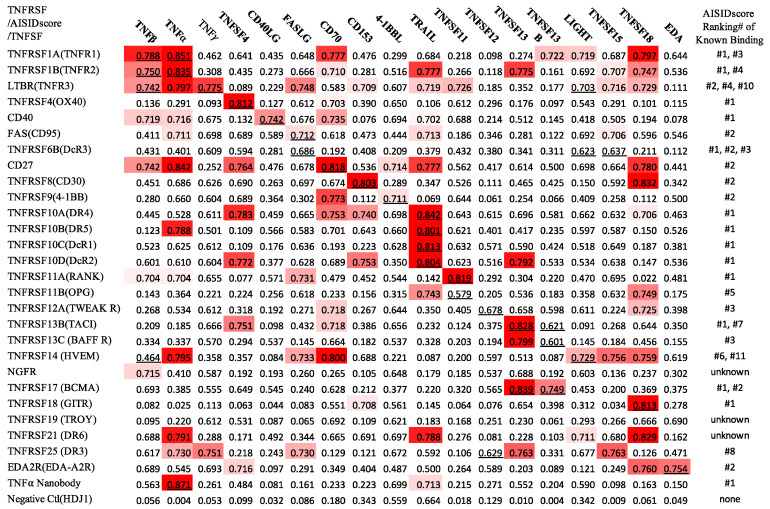
Heatmap of AISIDscores from 29 bait proteins (27 TNFRSF members, TNFα nanobody for positive control, Hsp40Hdj1 for negative control) against 18 TNFSF members. The AISIDscore of correct pairings between bait protein and TNFSF members are underlined. For each TNFRSF member, AISIDscores were ranked among 18 TNFSF members, with the ranking positions of the correct pairs are listed at the rightmost column. Three TNF receptor (NGFR, TROY, and DR6) do not have known ligands in TNFSF members and are labeled as “unknown”. The human Hsp40Hdj1 is labeled as “none”.

To test our method, 27 human TNFRSF members and 18 human TNFSF members were utilized. In all, 24 TNFRSF members out of the 27 can specifically bind at least one of the 18 TNFSF members as documented by the Uniprot database [15]. The specific binding between a TNFRSF member and its ligand TNFSF member(s) leads to distinct signaling pathways, including cell apoptosis, inflammation, and proliferations [16,17]. In each test, two protein sequences, one from a TNFRSF member and the other from a TNFSF member, were input into the AlphaFold-Multimer. The whole screening test generated a panel of AISIDscores for 27 × 18 pairs of TNFRSF and TNFSF members. For each TNFRSF member (as a bait protein), we ranked the AISIDscore among the 18 TNFSF members. The correct pairing was revealed as the highest AISIDscore for 13 out of 24 TNFRSF members, which have known specific interactions with TNFSF members. Out of the 33 correct pairings between TNFRSF and TNFSF members, 28 pairs could be found in the top five (including 25 pairs in the top three) seats of the AISIDscore rankings (Figure 1). The AISIDscore ranges from 0.02 to 0.85 with an average value of 0.46 for all pairs, and a much higher average value of 0.74 for the correct pairs. As a negative control, the AISIDscores between Hsp40 Hdj1 and TNFSF members were also calculated, resulted in a much lower average value of 0.15.

**Figure 2:**
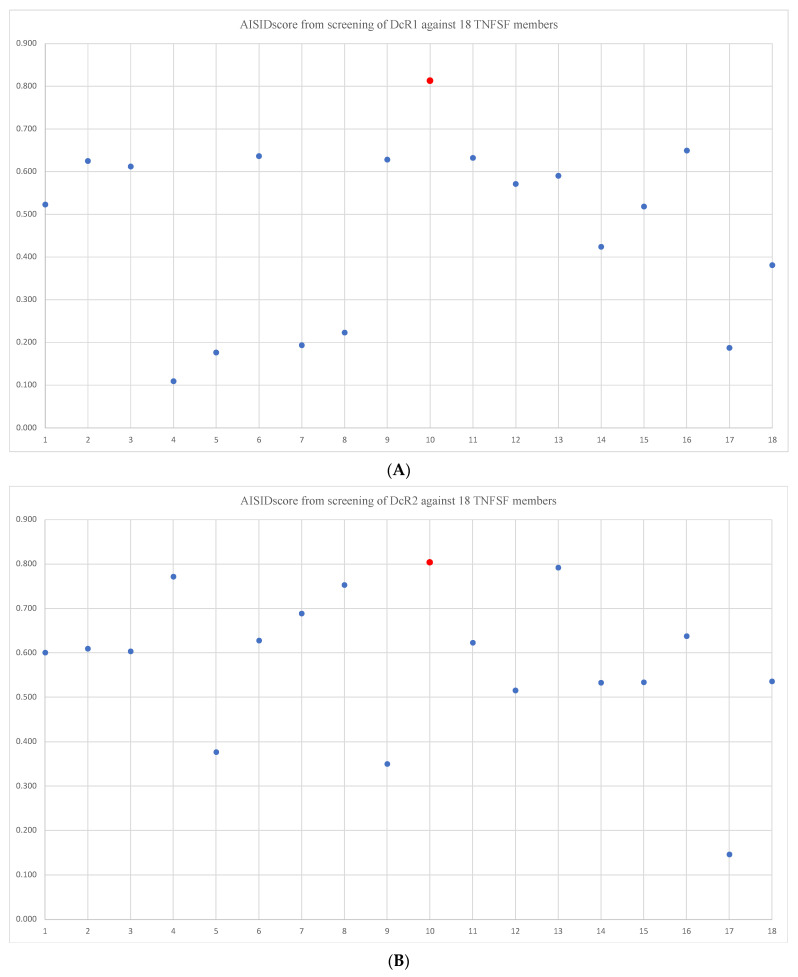

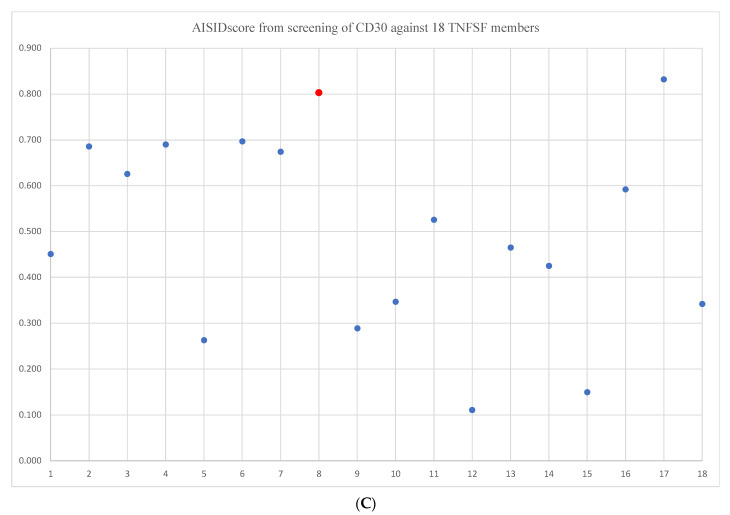
Plot of AISIDscores from screening the TNFRSF members, which do not have solved complex structures. (**A**–**C**), represent DcR1, DcR2, and CD30, respectively, with the 18 TNFSF members in same order as Figure 1. The red circle indicates the correct pairing.

It is of great interest to examine the ability of AISID for specific interactions between proteins with unknown structures. The TNFRSF members TNFRSF10C and TNFRSF10D (TRAIL DcR1 and TRAIL DcR2) can interact specifically with their ligand TNFSF10 (TRAIL), while their structural information is missing. Surprisingly, the pairing between the DcR1 and DcR2 and their ligand TRAIL gave the highest AISIDscore in the list (Figure 1, Figure 2A,B). Biochemical studies have shown that both DcR1 and DcR2 could specifically bind TRAIL with the K_D_ of ~1nM. DcR1 and DcR2 do not interact with other TNFSF members [18,19]. In another case of CD30/CD30L, both CD30 and its ligand CD30L are with unknown structures. The prediction showed that the CD30/CD30L pair provided the second highest AISIDscore in the list (Figure 2C). The studies using Surface Plasmon Resonance(SPR) have indicated that CD30 specifically interacted with CD30L with the K_D_ of ~5 nM [20]. Therefore, the prediction results using the AISIDscore are consistent with the previous biochemical studies. The data clearly indicated that the AISIDscore derived from the AlphaFold-Multimer might be a useful indicator to identify the specific interactions.

As a positive control, we took advantage of a nanobody that can specifically bind TNFα [21]. The protein complex structure of TNFα and this specific nanobody was deposited into PDB on 13 October 2016. We asked the AlphaFold-Multimer to perform the complex structure prediction between the nanobody and 18 TNFSF members. To prevent the AlphaFold-Multimer using the structure information in PDB, we also perform the calculation with the cutoff date of 1 January 2016. In both cases, the calculations gave the highest AISIDscore for the correct pairing of TNFα and the nanobody (Table 1). These data strongly suggest that AISID may be utilized as a valid method for identifying novel specific protein bindings.

It is worth noting that a relatively high sequence homology exists in TNFRSF and TNFSF family members. For example, the TNFα and TRAIL share ~25% sequence identity and ~60% sequence similarity. Our data indicate that the AISID method can reveal the specific interactions even among the highly homologous TNFSF family members. It is highly likely that the AISID may greatly speed up the screening for unknown specific protein bindings by prioritizing a long list of potential binding partners. The AISID-guided biochemical assay may follow to confirm the bindings.

Many protein interactions are mediated by intrinsic disordered proteins (IDP). Some IDPs have been showed to undergo a disorder-to-order transition upon recognizing their physiological partners [22]. Trying to postulate the binding partners for IDP by examining its primary sequence would be extremely challenging. The AISID can put the IDP in the context with its potential partners to predict the binding, which may mimic the fold-and-bind scenario for IDP.

The detailed atomic structure of predicted complex model is not the focus of AISID screening. It is structure-blind, and no other prior biological information, except only a pair of protein sequences, are needed for calculating the AISIDscore. Users will not need to examine the accuracy of the model complex structures, which makes the method user-friendly for people working in wet lab.

The presented AISID method may have significant biological impact in general biochemistry and cell biology. To characterize a protein with unknown functions, it is important to identify its binding partners. Using the traditional methods such as a yeast two hybrid system has been shown to be problematic and time-consuming. The AISID method may provide a fast alternative to identify the possible binding partner for a bait protein from a long list of protein candidates. Our method offers an AISIDscore which quantitatively measures the likelihood for a particular protein-protein interaction. The data showed that the AISIDscore was accurate in revealing specific protein interactions even among highly homologous proteins. Such a method may revolutionize how protein interactions are characterized.

## 3. Methods

In this presented AISID method for specific protein bindings screening, the AlphaFold-Multimer [9] was used to calculate the ranking metric. The AlphaFold-Multimer is a publicly available software package (https://github.com/deepmind/alphafold, accessed on 18 March 2022) recently released by DeepMind which extends the AlphaFold2 to multiple chains during both training and inference with native support for multiple-chain featurization and symmetry handling, surpassing those of inference-only modifications to the AlphaFold2.

When used in complex structure prediction, the AlphaFold-Multimer generates a merit index, the model confidence, defined as a weighted combination of ipTM and pTM:

Model Confidence = 0.8 × ipTM + 0.2 × pTM(1)

which is used to assess the overall accuracy of a predicted complex structure model. The two weighted factors ipTM and pTM are for evaluating inter-chain and intra-chain quality, respectively. In cases when the predicted model with either one or both composite proteins does not have high accuracy, the ‘Model Confidence’ thus defined could skew the evaluation of specific bindings, especially when not all domains of the proteins folded meaningfully. For identifying the potential specific binding between a bait protein and a ligand protein, a metric using only the inter-chain part would be more appropriate. In heterodimer complex structure predictions, the AlphaFold-Multimer generates 25 models by default, each with a model confidence. Due to the reasons described above, we derive a new metric (denoted as AISIDscore for simplicity) to assess binding potential between the proteins pair:

AISIDscore = max{1.25 × (Model Confidence) − 0.25 × avp}(2)

Here, ‘avp’ is the averaged percentage pLDDT [1] score of all amino acid residues in the predicted complex structure model, and ‘max{}’ runs through the 25 models to calculate the maximum. The AISIDscore thus defined is intended to highlight binding potential assessment by weighting down the impact of possible folding errors of individual chains.

The sequence of the bait protein to be investigated, and each sequence from the list of candidate proteins to be screened, are used to create a composite sequence file as input to the AlphaFold-Multimer for heterodimer complex structure predictions. As computing time of a prediction job heavily depends on the size of sequence, the list of composite sequence files is sorted by the number of amino acid residues from small to large. The sorting helps optimize the project production cycle (not the machine time of a prediction job), especially when carrying out a large screening project on a server with limited computing nodes and/or the candidate proteins are quite different in sizes. Batch jobs with inputs from the sorted list of composite sequence files are submitted through a load-balanced scheduler to the computer server running the AlphaFold-Multimer. Each of the composite sequence file will instruct the AlphaFold-Multimer to generate 25 (by default) predicted heterodimer complex structure models. The AISIDscore is then derived for the pair from harvesting and analyzing the outputs. Upon the finish of the screening project, a list of AISIDsocres will be generated, each for one bait-ligand pair.

For proof of concept, we picked 29 bait proteins including 27 human TNFRSF (tumor necrosis factor receptor superfamily) member proteins, a TNFα nanobody as positive control, and protein Hsp40 Hdj1 as negative control. The 18 human TNFSF (tumor necrosis factor superfamily) member proteins are used as the ligand proteins to be screened. Human TNFRSF and TNFSF are two of the well-studied protein superfamilies with many known specific bindings, which is good for methods validation and evaluation. Figure 1 shows the results from the 29 screening projects.

**Figure 3:**
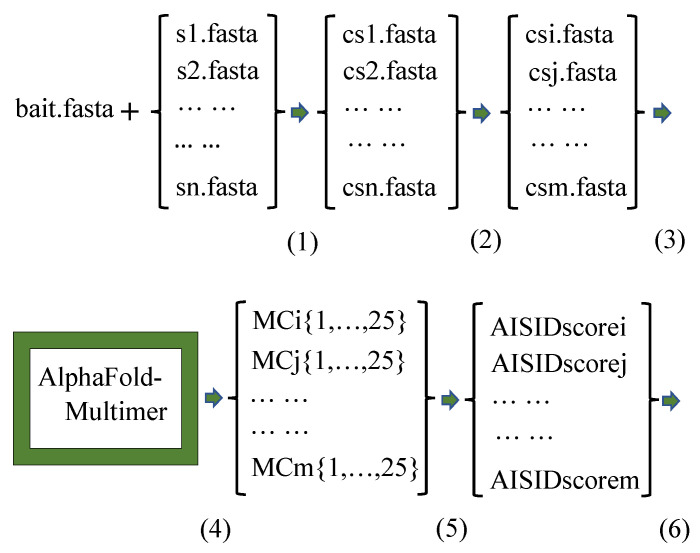
Workflow diagram of specific protein binding screening: (**1**). Create a list of composite sequence files of FASTA-format. Each file contains two separate sequences, one is that of the bait protein, the other is from one of the ligand proteins against which to be screened. (**2**). Sort the list by the number of amino acid residues in the composite sequence files from small to large. (**3**). Through a load-balanced scheduler, submit the batch jobs onto the server running the AlphaFold-multimer. (**4**). Once the project finishes, each job generates 25 predicted heterodimer complex structure models. (**5**). Harvest outputs and calculate the AISIDscore for each bait-ligand pair, resulting in a list of AISIDscores for the screening project. (**6**). Sort the list by AISIDscore from large to small for evaluation.

The AISID screening focuses on finding the potential specific binding proteins, not the detailed atomic structure of the predicted complex models. The sequences of bait and ligand proteins are the only inputs to the AlphaFold-Multimer for calculating the the AISIDscores. No other prior biological information is needed. For routine uses, one simply needs to examine the AISIDscore in search for the potential specific binding protein(s). The workflow diagram of a typical screening project is summarized in Figure 3. A group of in-house helper scripts have been created to facilitate executing the steps. From sequences to the ranked list of final AISIDscores, a screening project could be carried out by wet-lab scientists without special training in modeling, nor in interpreting details of an atomic structure.

## 4. Conclusions

In our test sets, 27 human TNFRSF members and 18 human TNFSF members were utilized. The specific interactions between TNFRSF members and TNFSF members have been extensively studied. Relatively high sequence homologues exist among TNFRSF members and TNFSF member, while each TNFRSF member can specifically bind TNFSF members to initiate distinct signaling pathways. The interaction network constituted by TNFRSF and TNFSF members provide us with an excellent database to investigate the AISID method for revealing specific protein bindings. We reason that this small but very well-informed database may be ideal for testing our method. Our results showed that out of the 33 correct pairings between TNFRSF and TNFSF members, 25 pairs can be found in the top three seats of the AISIDscore rankings (Figure 1).

The data in this study strongly suggest that the AISID method has the capability to identify novel protein interactions. It may greatly speed up the screening for unknown specific protein bindings by prioritizing a long list of potential binding partners. The AISID method may have broad applications for protein characterizations in protein biochemistry, extending the application of the AlphaFold far beyond structure predictions.

## Figures and Tables

**Table 1:** AISIDscores of TNFα nanobody against TNFSF superfamily.

TNFSF/AISIDscore	Default	Cutoff Date 1 January 2016
TNFα	0.871	0.869
TRAIL	0.713	0.705
4-1BBL	0.699	0.392
LIGHT	0.590	0.709
TNFβ	0.563	0.515
TNFSF13	0.552	0.454
TNFSF4	0.484	0.118
TNFSF12	0.271	0.101
TNFγ	0.261	0.311
CD70	0.233	0.353
CD153	0.223	0.096
TNFSF11	0.215	0.265
TNFSF13B	0.204	0.253
TNFSF18	0.163	0.276
FASLG	0.161	0.155
EDA	0.150	0.158
TNFSF15	0.098	0.089
CD40LG	0.081	0.082

## Data Availability

Not applicable.
